# A multicenter, retrospective, cross-sectional analysis of fresh and processed *Vitis vinifera* (grape) ingestion in dogs and cats

**DOI:** 10.3389/fvets.2026.1812885

**Published:** 2026-08-19

**Authors:** Kara D. Altman, Tanvi A. Kamat, Lucy Leicester, Tim Fraser, David A. Singleton, Neus Elias Santo-Domingo

**Affiliations:** 1 Emergency and Critical Care Department, Vets Now 24/7 Pet Emergency Specialty Hospital, Glasgow, United Kingdom; 2 Vets Now Limited, Dunfermline, United Kingdom; 3 IVC Evidensia Research United Kingdom, The Chocolate Factory, Bristol, United Kingdom

**Keywords:** activated charcoal, emesis, fluid therapy, grape, intoxication, nephrotoxin, raisin, *Vitis vinifera*

## Abstract

**Introduction:**

*Vitis vinifera* fruits are common household toxicants and domestic pets are often presented to veterinarians following exposure due to risk of acute kidney injury.

**Objective:**

To develop a regular expression–based method for identifying cases of *Vitis vinifera* fruit ingestion in electronic health records and to use this dataset to compare outcomes between fresh and processed exposures in canines and felines. Secondary aims were to characterize differences in outcome by species, demographics, seasonality, chocolate co-ingestion, and treatment patterns to inform risk stratification and management.

**Methods:**

A retrospective cross-sectional analysis was performed using emergency-care electronic health records from 64 United Kingdom–based private veterinary emergency centers between 2018 and 2024. Iteratively refined Python-based regular expressions were developed and validated to identify suspected or confirmed ingestion from free-text clinical narratives. Cases with confounding toxicities or incomplete records were excluded. Demographic, treatment, and outcome data were extracted. Mortality analyses were conducted by species with correction for multiple comparisons. Decision tree models were developed for canines and felines using stratified training and testing datasets with Bayesian hyperparameter optimization. Model performance was assessed using average precision on held-out data.

**Results:**

31,425 probable canine cases and 1,030 probable feline cases were identified. Processed fruit ingestion was more common than fresh fruit ingestion in both species (canines: 61.6% versus 31.2%; felines: 65.0% versus 31.1%), with seasonal clustering in December (canines: 22.0%; felines: 15.5%). Mortality was 1.3%, significantly higher in felines (11.8%) than in canines (0.9%). Fresh fruit ingestion was associated with higher mortality in both species (canine relative risk 3.47; feline relative risk 3.13). Age was associated with mortality in both species, and increased weight was associated with mortality in canines only. Chocolate co-ingestion was not associated with increased mortality. Treatment differed by species, with emesis more common in canines and intravenous fluids more frequent in felines. Decision tree analysis identified decontamination as the strongest predictor of survival.

**Clinical significance:**

*Vitis vinifera* ingestion is common in canine emergency presentations and less frequent but more fatal in felines. Fresh fruit ingestion carries higher mortality risk in both species. Gastrointestinal decontamination was the variable most strongly associated with favorable outcomes and supports prompt, species-specific management strategies.

## 1 Introduction

*Vitis vinifera* fruits (VVF) are common household items frequently ingested by domestic pets (1). These can be categorized as fresh VVF (FVVF), such as grapes, or processed VVF (PVVF), including raisins, currants, and sultanas. In 2001, the first retrospective study established an association between canine VVF ingestion and the development of acute kidney injury (AKI) (2). Since then, several further retrospective studies have detailed VVF ingestion and intoxication cases, many of which were asymptomatic (3–11). Symptoms, when present, are predominately gastrointestinal, though neurological and hematological signs have also been reported (10, 11).

The toxic mechanism resulting in kidney injury remains unknown (10). Histopathological analysis of kidneys from affected canines reveals renal tubular necrosis, particularly affecting the proximal tubules, with epithelial cell sloughing and basement membrane stripping (12). Similar renal histopathological changes have been observed in canines developing AKI secondary to tartaric acid exposure, and given the presence of tartaric acid in VVF, this currently represents the most widely suspected etiological factor (10, 13, 14). Notably, canines lack renal expression of the efflux transporter OAT-4, and experimental work in canine kidney cells has shown that inhibition of OAT-1 with probenecid significantly reduces tartaric acid–induced cytotoxicity, supporting a transporter-mediated mechanism for toxin accumulation and proximal tubule injury (14). To the authors’ knowledge the toxic mechanism has not been elucidated in felines.

The primary goal of treating VVF exposure is to prevent AKI; however, the unidentified mechanism of toxicity has led to considerable variation in clinical recommendations and management. The minimum toxic dose, if one exists, remains unknown, and it is unclear whether toxicity differs significantly between FVVF and PVVF. Plausible differences in toxicokinetics between FVVF and PVVF may mean current decontamination and monitoring protocols are insufficient or excessive in the face of individual intoxication.

The Veterinary Poisons Information Service (VPIS) currently recommends decontamination for all exposures using gastric emptying and activated charcoal followed by treatment with intravenous fluid therapy (5, 8). Nonetheless, substantial variability persists in diagnostic and therapeutic approaches (10). Notably, despite only 28.3% of cases being managed according to VPIS guidance, the largest retrospective study to date, published in 2021, reported that just 1 in 606 (0.001%) canines developed AKI (8).

A 2024 scoping review identified 24 studies documenting a total of 2,073 canine cases of VVF ingestion (10). However, to the authors’ knowledge, no previous study has examined clinical differences between exposures to FVVF versus PVVF, representing a significant gap in the current understanding of this condition. In addition, there is a notable paucity of data regarding feline VVF exposure, with one report documenting two felines that developed fatal acute renal failure following ingestion (5) and another documenting 13 felines exposed to VVF where two developed clinical signs and none developed AKI (9). This highlights the potential for species-specific differences in susceptibility and clinical outcomes. It remains unclear whether feline cases are under-represented due to lower exposure rates, increased sensitivity, or under-recognition due to non-specific clinical signs or unwitnessed ingestion. Consequently, current treatment recommendations for feline VVF exposure are extrapolated from canine data.

A further complicating factor in the clinical picture of VVF exposure is the frequent co-ingestion of chocolate, given the common pairing of these substances in commercial products (6). Noble et al. (2017) reported three cases of combined chocolate and PVVF ingestion in which the combination did not appear to affect disease outcome (15). Nonetheless, the dose-dependent cardiac and neurological effects of chocolate toxicosis raise the theoretical possibility that co-ingestion could compound VVF-associated renal and gastrointestinal injury, a question that has not been systematically addressed in the existing literature (1, 16).

The aim of this study was to develop a regular expression to search electronic health records from a nationwide network of private veterinary emergency clinics and hospitals, thereby identifying a large cohort of VVF exposure cases encompassing both canine and feline patients to compare outcomes between FVVF and PVVF exposures, with and without chocolate co-ingestion and to describe patterns in species difference, treatment, seasonality, and mortality. By leveraging novel data extraction methods to create a substantially larger dataset than previous studies, this work aims to build on existing knowledge of VVF toxicity, provide practical insights to inform clinical decision-making, and help guide the most effective directions for future research.

## 2 Materials and methods

### 2.1 Regular expression formation

Veterinary electronic health records (EHRs) from 2018 to 2024 were obtained from a nationwide network of 64 private veterinary emergency centers in the United Kingdom providing out-of-hours care for more than 1,700 primary care practices (17), with all clinical information recorded and stored using the practice management system Helix®.

To develop and validate regular expressions (regexes) and identify candidate cases with VVF exposure, the Helix database was queried using structured query language via Microsoft Server Management Studio (18), creating a randomized sample of 10,000 EHRs annually from 2018 to 2024. The final sample of 70,000 EHRs comprised a representative mix of attending canine and feline cases, which proportionally reflected the species distribution in the overall emergency caseload.

Cases were identified based on suspected or confirmed VVF ingestion recorded in the free-text clinical narrative by the call handler or attending veterinary professional. Ingestions were classified as FVVF if involving grapes, and as PVVF if involving processed products or foods containing these (e.g., fruit cake).

Potential cases were initially screened using Python-based regexes (19), separately developed for FVVF and PVVF and refined for differences between phone-call and consultation notes. After applying the VVF regexes, an additional regex was used to detect co-ingestion of chocolate, with flagged cases reviewed to confirm both VVF and chocolate exposures. Regexes were iteratively refined using random samples of 500 consultations per iteration—100 reviewed by both authors (KA, TK) and 200 individually—to capture common phrases, spelling variations, inclusion terms, and frequent negations (e.g., canines named after VVF or chocolate products, historical ingestion, toxin lists, or owner-confirmed non-ingestion), continuing until true positives predominated.

The final regexes were applied to 973,723 unique feline and 1,330,021 unique canine cases. Across phone-call records, 986 cases were manually reviewed (500 positive and 486 negative cases per regex), while an analogous sampling strategy was applied to consultation notes (500 positive and 500 negative cases per regex). Cases flagged for co-ingestion of chocolate were reviewed concurrently with VVF cases to confirm both exposures. Performance metrics were calculated across the four groups, phone-call-FVVF, phone-call-PVVF, cons-note-FVVF, and cons-note-PVVF, with all regexes demonstrating excellent performance (see Table 1).

**Table 1:** Performance metrics for regexes.

Regex	Sensitivity	Specificity	Accuracy	Recall	Precision	F1	Cohen’s Kappa
Phone-call FVVF	1	0.98	0.99	1	0.98	0.99	0.99
Phone-call PVVF	1	0.98	0.99	1	0.98	0.99	0.98
Cons-note FVVF	1	0.87	0.93	1	0.85	0.92	0.93
Cons-note PVVF	1	0.94	0.97	1	0.93	0.96	0.93

Review of false positives identified frequent misclassification between FVVF and PVVF (60/125 cases); prompting introduction of a third category, unknown VVF ingestion (UVVF), to account for conflicting or overlapping evidence across records. The final regex identified 10,119 FVVF and 20,035 PVVF cases and a further 2,301 cases were classified as UVVF according to the above definition. Final regexes are accessible in Supplementary material.

### 2.2 Grouping and filtering of data

**Figure 1:**
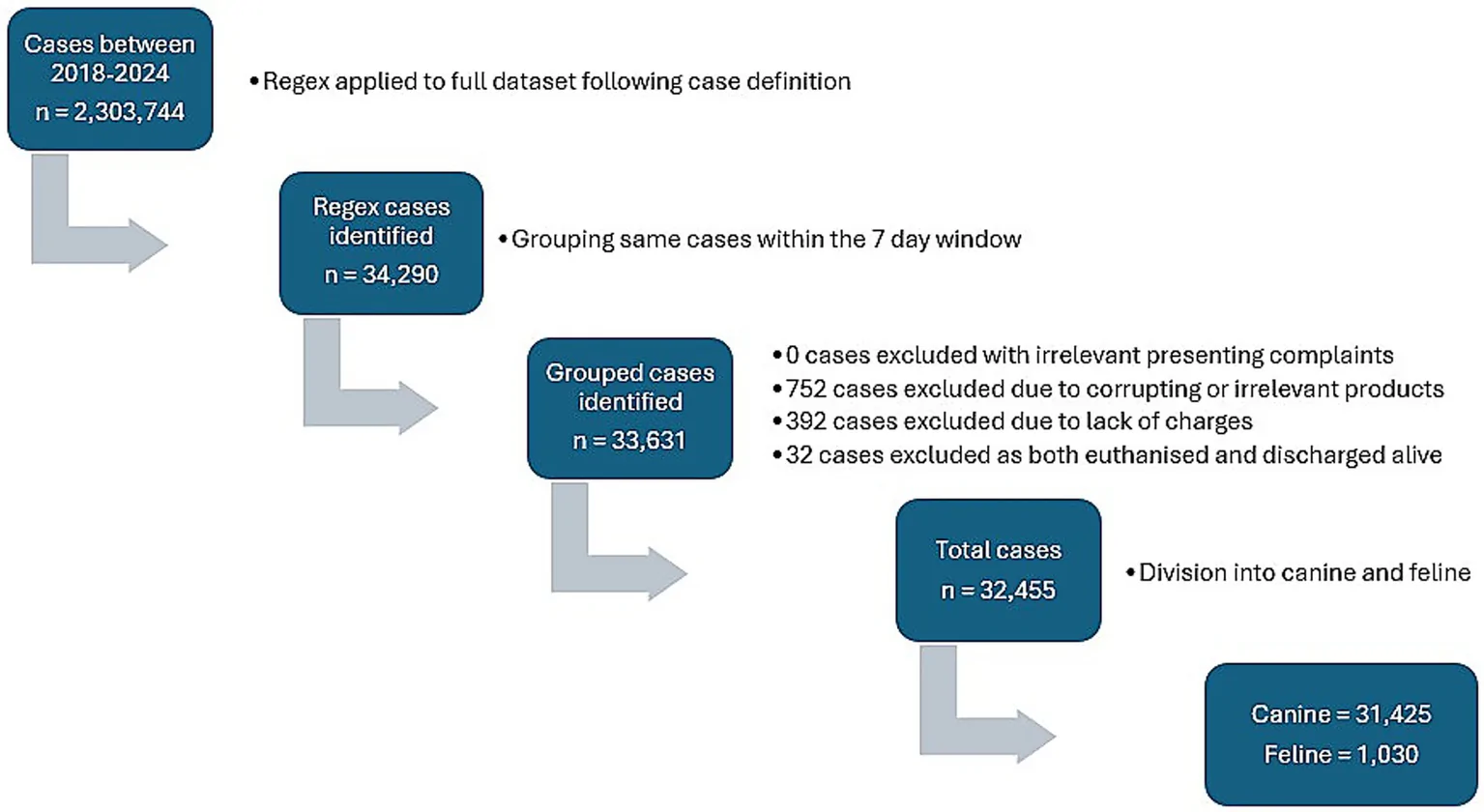
Iterations of regex.

Resulting case data was initially analyzed in Microsoft Excel® v365 (20). Python (19) was used to group events into seven-day windows; exclude cases with irrelevant products; cases with no products; cases with conflicting discharge statuses (i.e., alive and dead); and identify multi-case animals in that order. See Figure 1 for regex iteration process.

Each case included a presenting complaint, typically recorded by the attending veterinarian at the time of consultation. When cases were grouped into seven-day windows, only the first complaint was kept. A review of the presenting complaints confirmed that all were aligned with the anticipated signs or clinical manifestations of VVF ingestion.

Treatments, monitoring and clinical events were categorized as emesis, anti-emetics, activated charcoal, blood tests, intravenous fluid therapy, hospitalization, urethral catheterization, consumables (products used for urethral catheterization), furosemide and euthanasia/death. Categories were defined by compiling all chargeable items and assigning them accordingly. Cases were excluded if charges indicated irrelevant or confounding conditions (e.g., the use of other potential nephrotoxins such as non-steroidal anti-inflammatories) resulting in removal of 752 cases. An additional 392 cases were excluded due to absence of any charges.

Mortality was determined from both discharge status and euthanasia/death charges, with 32 cases excluded due to conflicting records (both euthanized and discharged alive). Cases with either euthanasia/death charges or a discharge status of “died” were counted once, with euthanasia and unassisted death combined for all future analyses.

Cases were stratified by weight into small (<10 kg), medium (10- < 25 kg), large (25–<40 kg), giant (≥40 kg) or unknown categories, using recorded weight, or breed-specific average weight when individual data was unavailable. Breed listings such as crossbreed, terrier and hound were classified as unknown. Age was categorized as 0–2 years, >2–5 years, >5–8 years, >8–11 years, >11 years or unknown however, age was entered as a continuous variable, rather than the categorized form, for decision tree analysis.

The result from the review of the retrieved dataset was deemed sufficiently accurate, such that all retrieved cases were included as probable cases in further analysis and modelling. The regex retrieved a total of 31,425 probable canine and 1,030 probable feline cases following exclusions.

### 2.3 Statistical testing

All analyses were conducted using Python v3.12 (19). Statistical analyses were performed using the *statsmodels* (v0.14.5) and *scipy* (v1.16.2) libraries. Decision tree models were developed and evaluated using *scikit-learn* (v1.7.2), and hyperparameter optimization was conducted using *Optuna* (v4.5.0). Data manipulation and preprocessing were performed using *pandas* (v2.2.3) and *NumPy* (v2.2.4).

For mortality analysis, to satisfy the assumption of independence between observations and groups, only the final ingestion event (in multi-event animals) was included in statistical testing. As a result, testing data for mortality contained 32,049 cases, 31,024 canine and 1,025 feline. An initial chi-squared (χ^2^) test for independence demonstrated significant association between species and death. Given this strong species-level effect, all subsequent statistical analyses were conducted separately and species-specific decision tree models were developed. In contrast to mortality analysis, decision tree models retained repeated ingestion events as separate cases to maximize available information for pattern discovery as this does not require independence between observations.

Within species, differences in the distribution of selected qualitative variables were assessed using the χ^2^ test, Fisher’s exact test, or the Cochran–Armitage test for trend, as appropriate. A Benjamini–Hochberg correction was applied to account for multiple comparisons, and an adjusted *p*-value < 0.05 was considered statistically significant.

### 2.4 Decision tree model development and evaluation

For each species, total data were divided into training (85%) and testing (15%) sets using stratification on mortality to maintain comparable outcome proportions across splits (see Table 2). The testing dataset was held out from all model development steps and used only for final performance evaluation.

**Table 2:** Division of data into training and testing sets.

Canine	Raw cases	Positives (euth/death) cases	% Positives
Training set data	26,711	251	0.940
Testing set data	4,714	44	0.933
Total	31,425	295	0.939

Feline	Raw cases	Positives (euth/death) cases	% Positives
Training set data	875	104	11.886
Testing set data	155	18	11.613
Total	1,030	122	11.845

Because death was a rare outcome, class weighting was applied during model fitting so that survival and death contributed equally to the training objective. Consequently, both model-derived (weighted) probabilities and empirical (raw) probabilities are reported and interpreted where appropriate.

Decision tree hyperparameters were optimized using Bayesian optimization implemented in *Optuna*. A total of 400 optimization trials were performed for felines and 600 for canines, with each evaluating a unique combination of hyperparameters defining tree structure. Model performance during optimization was assessed using stratified k-fold cross-validation on the training data (*k* = 5 for felines; *k* = 3 for canines). For each trial, mean average precision for the death class was calculated across folds.

After completion of all trials, the best-performing hyperparameters were used to refit a final model on the full training dataset. This optimized model was then evaluated on the held-out testing dataset, with test-set average precision providing the primary estimate of model generalizability.

## 3 Results

### 3.1 Patient demographics

The age, weight and sex distribution of the 32,455 cases included is summarized in Table 3.

**Table 3:** Summary of case demographics and mortality.

Case demographics	Canine		Feline	
	Total (% of total 31,425)	Mortality (% of category)	Total (% of total 1,030)	Mortality (% of category)
Age in years				
0–2	13,592 (43.3%)	32 (0.2%)	348 (33.8%)	17 (4.9%)
>2–5	8,429 (26.8%)	27 (0.3%)	193 (18.7%)	19 (9.8%)
>5–8	4,437 (14.1%)	33 (0.7%)	132 (12.8%)	16 (12.1%)
>8–11	2,413 (7.7%)	62 (2.6%)	126 (12.2%)	18 (14.3%)
>11	1,241 (3.9%)	122 (9.8%)	155 (15.0%)	37 (23.9%)
Age not recorded	1,313 (4.2%)	19 (1.4%)	76 (7.4%)	15 (19.7%)
Weight in kilograms				
Small (<10)	9,164 (29.2%)	79 (0.9%)	399 (38.7%)	34 (8.5%)
Medium (10– < 25)	10,886 (34.6%)	90 (0.8%)	4 (0.4%)	1 (25.0%)
Large (25– < 40)	6,238 (19.9%)	61 (1.0%)	-	-
Giant (40 or above)	485 (1.5%)	18 (3.7%)	-	-
Unknown	4,652 (14.8%)	47 (1.0%)	627 (60.9%)	87 (13.9%)
Sex				
Male	15,718 (50.0%)		592 (57.5%)	
Female	13,742 (43.7%)		365 (35.4%)	
Sex not recorded	1965 (6.3%)		73 (7.1%)	

Most patients were young, with 43.3% of canines and 33.8% of felines aged between 0 and 2 years. Males were overrepresented in both species (50.0% canines and 57.5% felines). Among canines, small and medium-sized canines were the most frequent (63.8%).

Canine breeds that were represented by >500 cases were as follows: Labrador Retriever (3,058; 9.7%); Cockapoo (2,576; 8.2%); Cocker Spaniel (2,226; 7.1%); Labradoodle (747; 2.4%); French Bulldog (726; 2.3%); Golden Retriever (663; 2.1%); Miniature Dachshund (604; 1.9%); Border Collie (587; 1.9%); and Jack Russell Terrier (540; 1.7%).

Of the 1,030 feline cases, 380 (36.9%) were listed as domestic breeds (Domestic Short Hair, Domestic Medium Hair, Domestic Long Hair), 104 (10.1%) were listed as purebred breeds, and 546 (53.0%) were unknown. Feline breeds that were represented by > 15 cases were as follows: British Short Hair (24; 2.3%), Ragdoll (23; 2.2%), and Maine Coon (15; 1.5%).

### 3.2 Fresh (FVVF), processed (PVVF), unknown (UVVF) and chocolate ingestion

**Figure 2:**
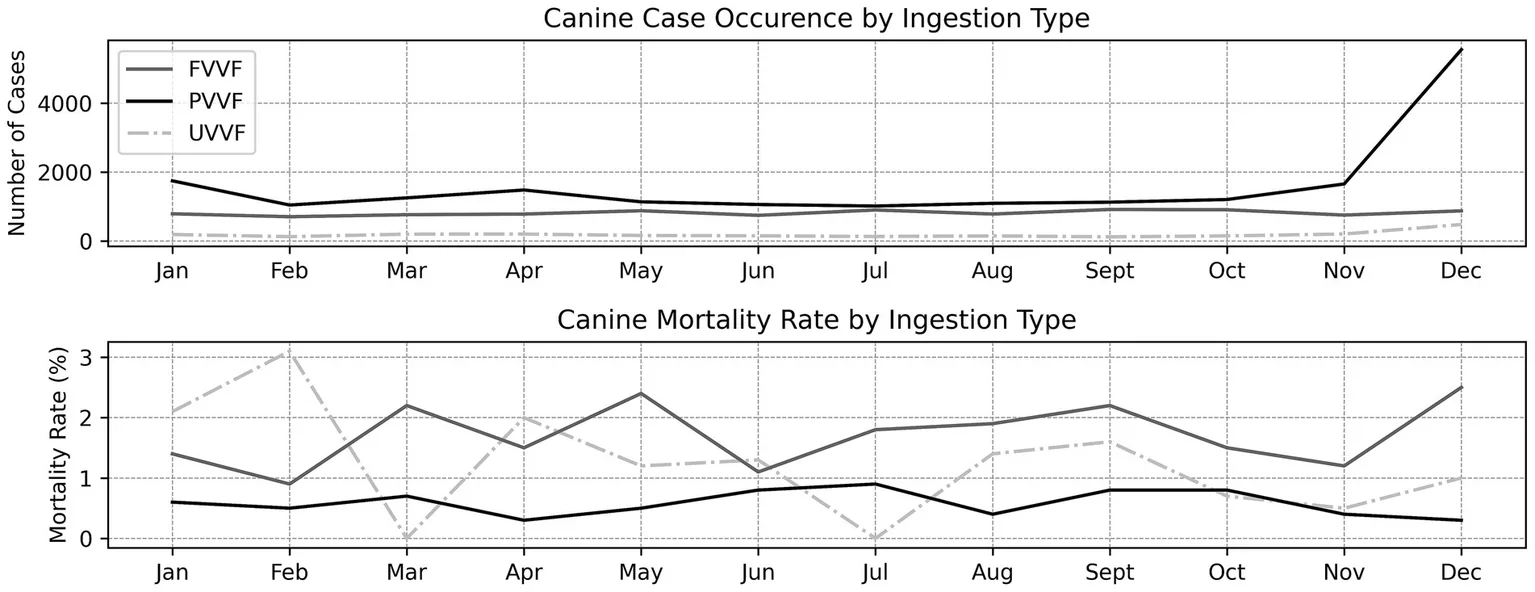
Canine monthly case occurrence and mortality rate by ingestion type.

**Figure 3:**
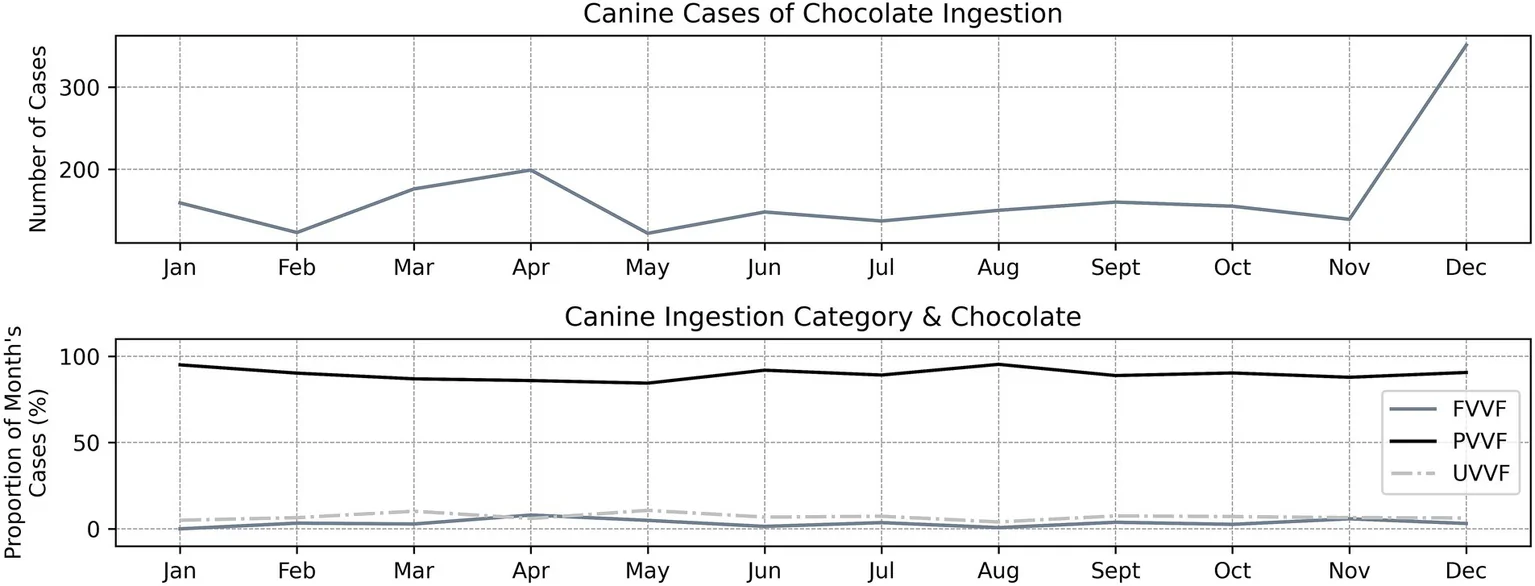
Canine co-ingestion with chocolate monthly case occurrence and ingestion type.

**Figure 4:**
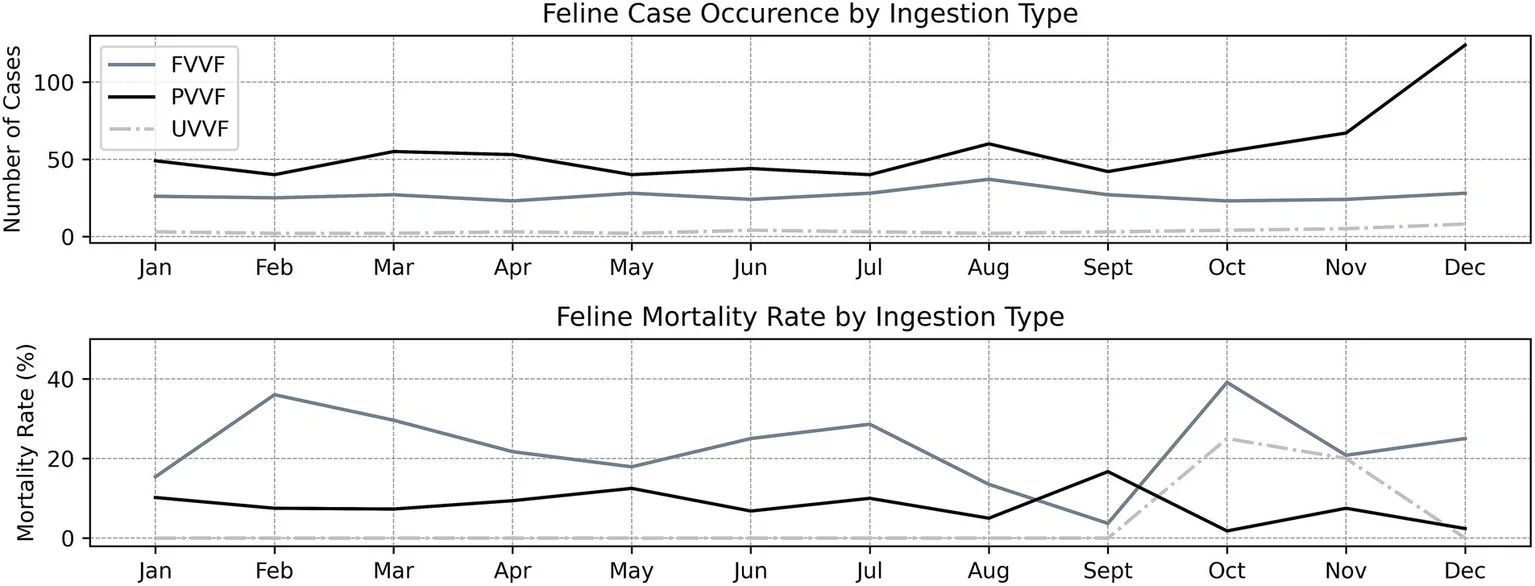
Feline monthly case occurrence and mortality rate by ingestion type.

**Figure 5:**
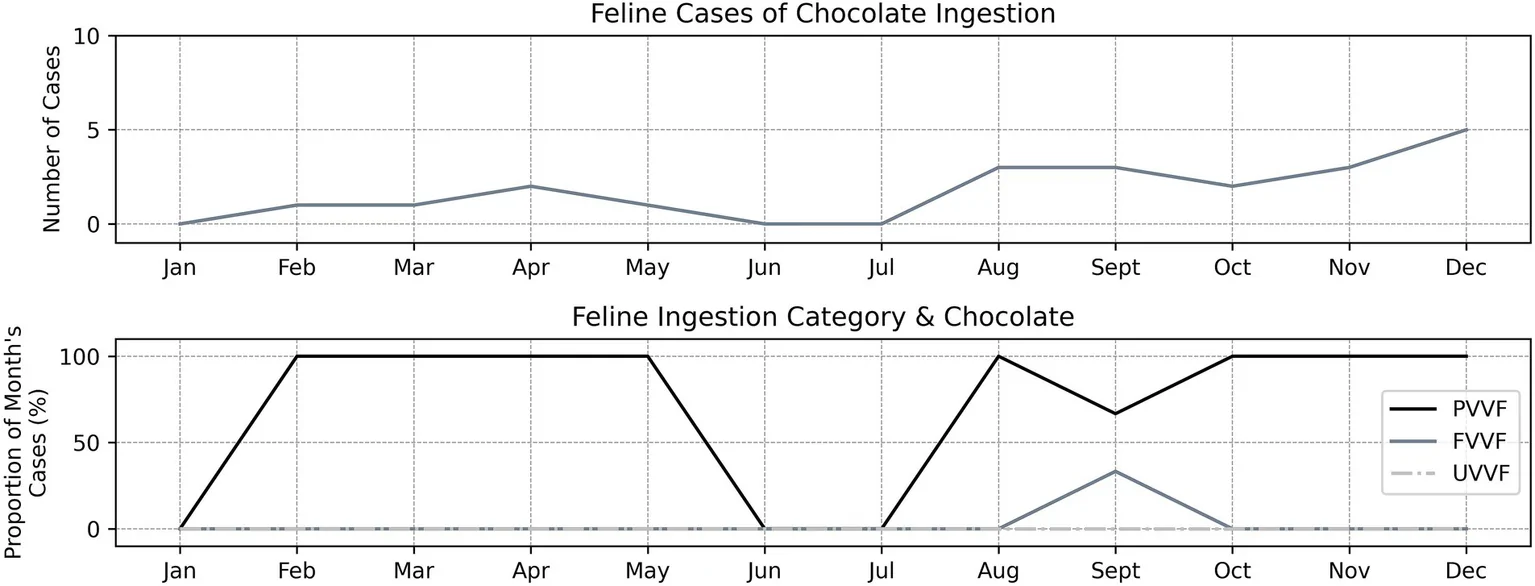
Feline co-ingestion with chocolate monthly case occurrence and ingestion type.

Summarized in Tables 4, 5 and Figures 2–5.

**Table 4:** Canine cases and treatment and monitoring categories.

Total cases 31,425	All cases (Percentage of total cases)	FVVF ingestion Total cases 9,799		PVVF ingestion Total cases 19,366		UVVF ingestion Total cases 2,260	
Categories		With Choc	Without Choc	With Choc	Without Choc	With Choc	Without Choc
Emesis	24,120 (76.8%)	52	7,077	1,558	13,652	121	1,660
Anti-emetic	8,333 (26.5%)	11	1,440	230	2,269	22	355
Emesis and anti-emetic	2,830 (9.0%)	2	860	181	1,548	21	218
Charcoal	18,982 (60.4%)	42	5,189	1,280	11,032	105	1,334
Fluid therapy	6,338 (20.2%)	10	1748	382	3,632	29	537
Furosemide	31 (0.1%)	0	13	0	15	0	3
Blood tests	9,522 (30.3%)	15	2,716	548	5,406	48	789
Hospitalization	14,144 (45.0%)	25	4,236	812	7,962	59	1,050
Urethral catheterization	29 (0.1%)	0	15	1	9	0	4
Emesis and charcoal	16,683 (53.1%)	35	4,468	1,134	9,772	93	1,181
Emesis, charcoal and blood tests	4,789 (15.2%)	8	1,102	375	2,838	36	430
Emesis, charcoal and fluid therapy	2,883 (9.2%)	5	678	239	1,672	23	266
Emesis, blood tests, fluid therapy and hospitalization	3,021 (9.6%)	5	711	238	1752	25	290
Fluid therapy and hospitalization	6,194 (19.7%)	10	1709	378	3,542	27	528
Mortality	295 (0.9%)	1	170	0	97	0	27

**Table 5:** Feline cases and treatment and monitoring categories.

Total cases 1,030	All cases (Percentage of total cases)	FVVF Ingestion Total cases 320		PVVF Ingestion Total cases 669		UVVF Ingestion Total cases 41	
Categories		With Choc	Without Choc	With Choc	Without Choc	With Choc	Without Choc
Emesis	327 (31.7%)	0	43	13	253	0	18
Anti-emetic	140 (13.6%)	0	51	0	80	0	9
Emesis and anti-emetic	18 (1.7%)	0	10	0	8	0	0
Charcoal	265 (25.7%)	1	17	13	216	0	18
Fluid therapy	368 (35.7%)	0	116	5	235	0	12
Furosemide	25 (2.4%)	0	8	0	16	0	1
Blood tests	473 (45.9%)	0	141	8	304	0	20
Hospitalization	493 (47.9%)	0	146	6	323	0	18
Urethral catheterization	24 (2.3%)	0	21	0	3	0	0
Emesis and charcoal	163 (15.8%)	0	6	9	138	0	10
Emesis, charcoal and blood tests	69 (6.7%)	0	1	3	61	0	4
Emesis, charcoal and fluid therapy	49 (4.8%)	0	0	2	44	0	3
Emesis, blood tests, fluid therapy and hospitalization	89 (8.6%)	0	20	4	62	0	3
Fluid therapy and hospitalization	341 (33.1%)	0	105	5	219	0	12
Mortality	122 (11.8%)	0	72	0	48	0	2

Across both species, PVVF ingestion was more common than FVVF. In canines, 19,366 (61.6%) cases involved only PVVF, compared with 9,799 (31.2%) FVVF cases, while 2,260 (7.2%) were classified as UVVF. In felines, 669 (65.0%) cases involved only PVVF, 320 (31.1%) involved only FVVF, and 41 (4.0%) were classified as UVVF.

A total of 2,019 (6.4%) canine and 21 (2.0%) feline cases also ingested chocolate as identified by the additional regex developed to identify co-ingestion. Co-ingestion with chocolate was more frequent in PVVF cases in both species, particularly in canines (1,812; 5.8% versus 68; 0.2% for FVVF and 139; 0.4% for UVVF) and to a lesser extent in felines (20; 1.9% versus 1; <0.1% for FVVF and 0; 0% for UVVF).

### 3.3 Temporal distribution

Temporal distribution is summarized in Figures 2–5.

There was a marked increase in canine cases seen in December (6,908; 22.0%) compared to other months (average 2,208; 7.1%; range 1,877-2,610; 6.0–8.3%). This trend was also seen in feline cases with cases in December (160; 15.5%) increased when compared to other months (average, 79; 7.7%; range 67–99; 6.5–9.6%). In both species this was most pronounced in PVVF cases. Cases that involved co-ingestion with chocolate were also most frequent in December across both species.

### 3.4 Multi-event animals

Three hundred ninety two canine and 5 feline animals ingested VVF multiple times during the study period, indicating repeated exposures. Together this totaled 803 events, and no multi-event animal died.

### 3.5 Treatment and monitoring

Treatments and monitoring are summarized in Tables 4, 5.

Treatment patterns differed between species. Emesis-inducing therapies were administered to 24,120 (76.8%) canine cases compared with 327 (31.7%) feline cases. Anti-emetics were given to 8,333 (26.5%) canine cases and 140 (13.6%) feline cases, with 2,830 (9.0%) canine cases and 18 (1.7%) feline cases receiving an anti-emetic as well as emesis induction.

Activated charcoal was more commonly administered to canine cases, at 18,982 (60.4%) compared to feline cases, 265 (25.7%). However, feline cases were more likely to receive fluid therapy (368, 35.7%), compared to canine cases (6,338, 20.2%). Furosemide was administered in 31 (0.1%) canine cases and 25 (2.4%) feline cases.

Blood tests were performed in 9,522 (30.3%) canine cases and 473 (45.9%) feline cases, while hospitalization occurred in 14,144 (45.0%) canine cases and 493 (47.9%) feline cases.

VPIS recommendations for treatment including induction of emesis and administration of activated charcoal and intravenous fluid therapy were followed in 2,883 (9.2%) canine and 49 (4.8%) feline cases.

Across both species, PVVF exposures consistently received more extensive treatment and monitoring, with higher frequencies of charcoal administration, blood testing, fluid therapy, and hospitalization.

The most common concomitant treatments included analgesia (4,038/32,455; 12.4%), antibiotics (1,011/32,455; 3.1%), sedatives (824/32,455; 2.5%) and gastroprotectants (789/32,455; 2.4%).

### 3.6 Mortality

Mortality is summarized in Table 3 and Figures 2, 4.

Overall, 295 (0.9%) canine cases and 122 (11.8%) feline cases died. Across all cases, this corresponds to a mortality rate of 1.3%, with annual mortality ranging from a low of 57/6,388 (0.9%) in 2023 to a high of 52/2,260 (2.3%) in 2018. No meaningful monotonic trends in annual mortality were observed.

A χ^2^ test for independence demonstrated a significant association between species and mortality (Benjamini-Hochberg (BH)-corrected *p* < 0.001). Felines exhibited substantially higher mortality risk than canines, with a conditional odds ratio (feline-to-canine) of 14.07 (95% confidence interval (CI): 11.18–17.62) and a relative risk of 12.52 (95% CI: 10.23–15.31).

Among canines, several variables were significantly associated with mortality outcomes. Males exhibited higher mortality (χ^2^ test; BH-corrected *p* = 0.024), although the magnitude of effect was small with conditional odds ratio (male-to-female) of 1.091 and a relative risk of 1.09 (95% CI: 0.856–1.387). Neuter status was not significantly associated with mortality (χ^2^ test; BH-corrected *p* = 0.198).

Ingestion type was strongly associated with mortality (χ^2^ test; BH-corrected *p* < 0.001). Canines ingesting FVVF exhibited higher mortality risk than those ingesting PVVF (χ^2^ test; BH-corrected *p* < 0.001), with a conditional odds ratio (fresh-to-processed) of 3.51 (95% CI: 2.72–4.56) and a relative risk of 3.47 (95% CI: 2.71–4.45). FVVF or PVVF consumed along with chocolate ingestion did not differ significantly from FVVF or PVVF ingestion alone.

Age was significantly associated with mortality (Cochran–Armitage trend test; BH-corrected *p* < 0.001), indicating a trend of increasing mortality with age. Weight was also significantly associated with mortality (Cochran–Armitage trend test; BH-corrected *p* = 0.007), suggesting higher mortality in heavier canines.

Among felines, neither sex nor neuter status demonstrated a statistically significant association with mortality outcomes (χ^2^ test, BH-corrected *p* = 0.621 and 0.105, respectively).

Ingestion type was significantly associated with feline mortality. Felines ingesting FVVF had a significantly higher risk of death compared with those ingesting PVVF (χ^2^ test, BH-corrected *p* < 0.001), with a conditional odds ratio (fresh-to-processed) of 3.74 (95% CI: 2.48–5.68) and a relative risk of 3.13 (95% CI: 2.23–4.40). FVVF or PVVF consumed along with chocolate ingestion did not differ significantly from FVVF or PVVF ingestion alone.

Age was significantly associated with mortality (Cochran–Armitage trend test; BH-corrected *p* < 0.001), indicating a trend of increasing mortality with age. Weight was not significantly associated with mortality (Cochran–Armitage trend test; BH-corrected *p* = 0.401).

### 3.7 Decision tree analysis and concordance between statistical testing and structure in canines

**Figure 6:**
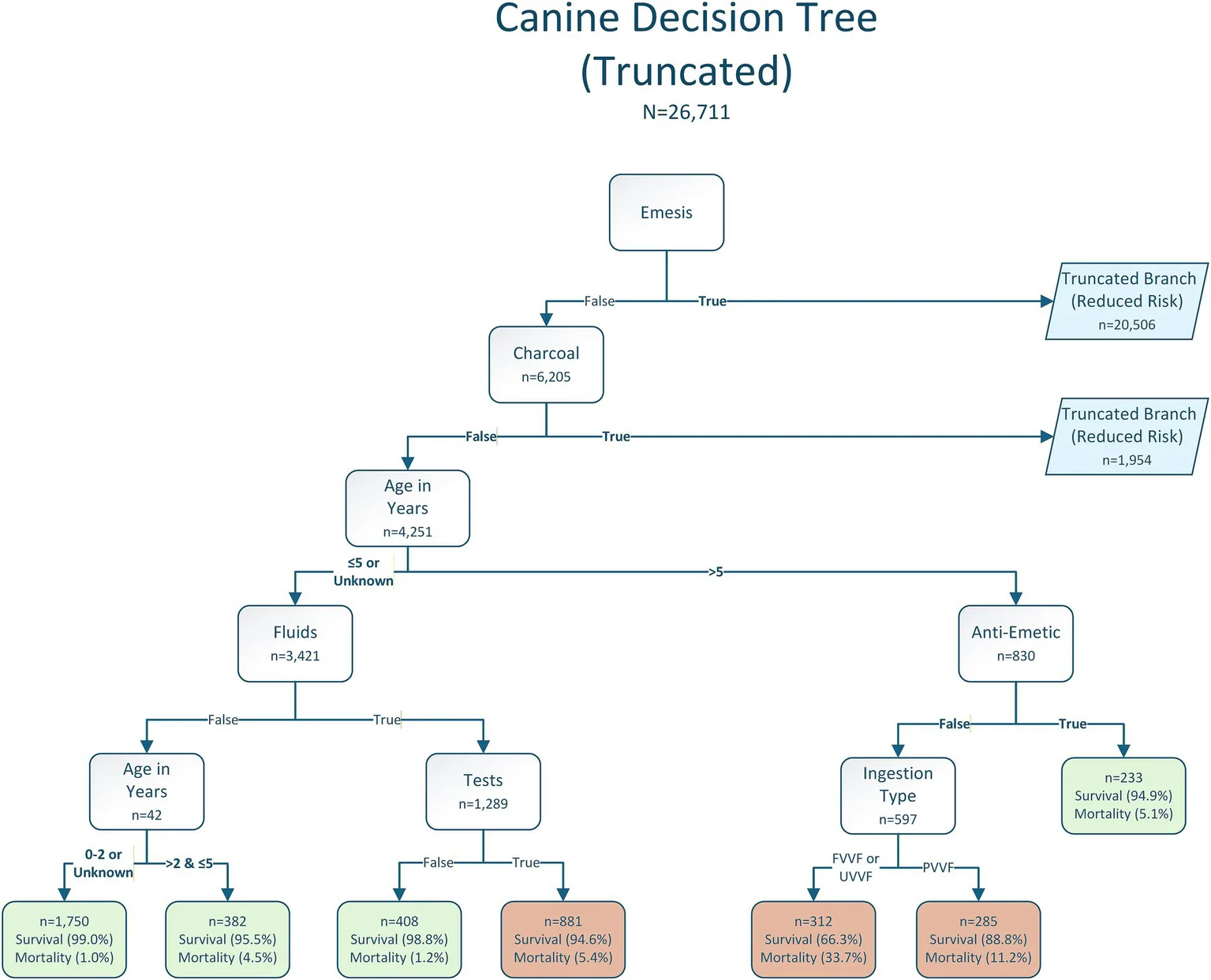
Canine decision tree.

See the canine decision tree in Figure 6.

Emesis status defined the primary split in the tree: canines that underwent emesis demonstrated uniformly high survival and comprised most cases. Canines without emesis represented a minority but accounted for a disproportionate share of mortality.

Within the no-emesis subgroup, outcome was significantly associated with additional treatment charges, particularly administration of activated charcoal and fluid therapy. Charcoal administration was the variable most strongly associated with survival within this subgroup.

Although age was statistically associated with outcome in canines, it functioned as a conditional modifier rather than a primary determinant of risk. Increased mortality among older canines (≥6 years) was predominant in the absence of additional treatment charges (emesis and charcoal), a pattern encoded in the decision tree structure. Ingestion type further refined risk only within these high-risk management pathways and lost importance if care was escalated.

Among younger canines without charcoal, fluid therapy and blood testing were associated with high survival (≥95%), whereas absence of treatment charges identified a higher-risk subgroup. Among older canines without charcoal, ingestion type became prognostically important. FVVF or UVVF ingestions were associated with the highest mortality (33.7%), whereas PVVF ingestions had lower mortality (11.2%), representing an approximately three-fold increase in risk of death.

Among canines that received emesis treatments, associations persisted with age, weight/breed, sex, and season; however, survival remained uniformly high. Decision tree splits within this pathway reflected population heterogeneity rather than clinically meaningful risk differentiation.

### 3.8 Decision tree analysis and concordance between statistical testing and structure in felines

**Figure 7:**
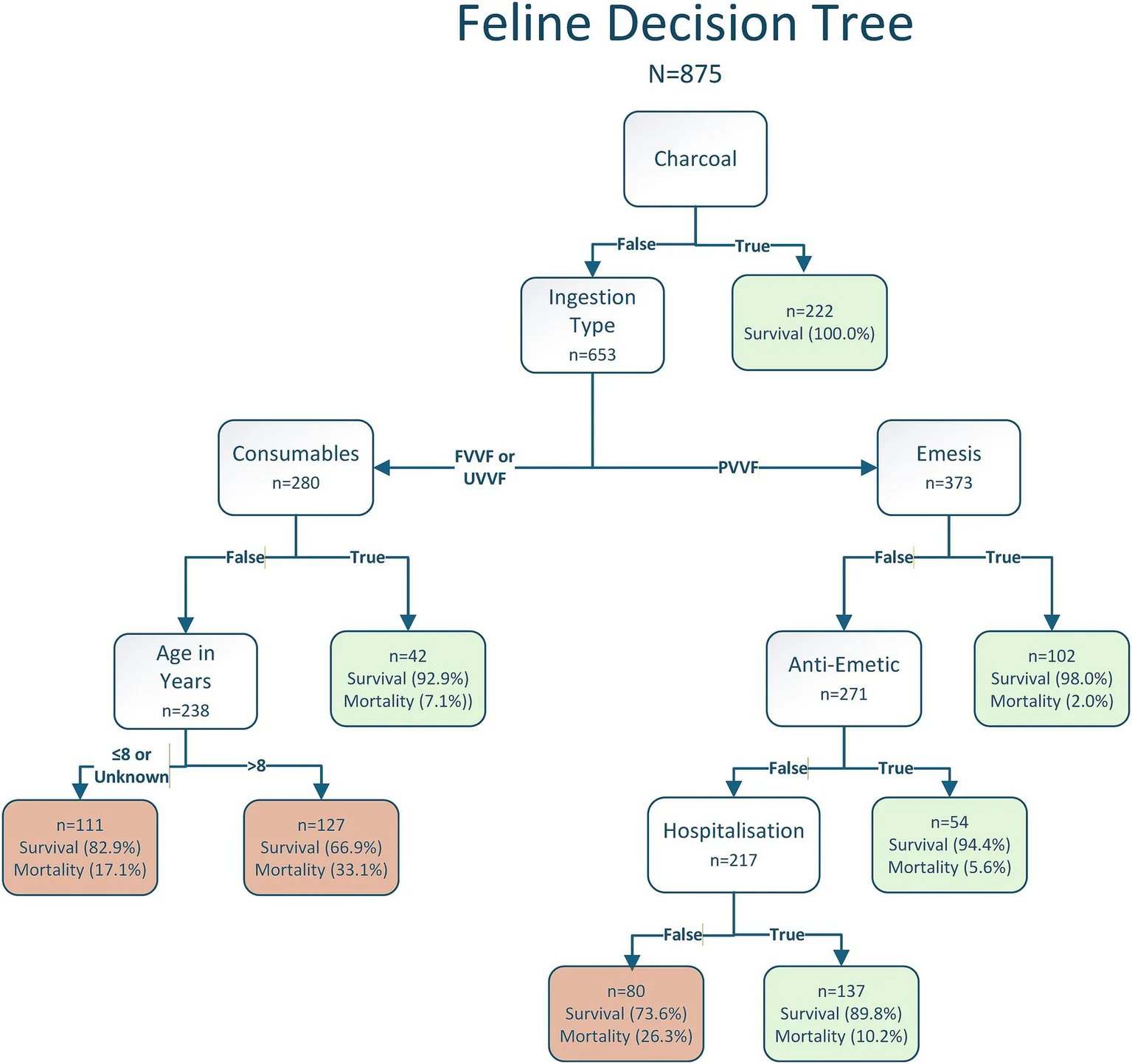
Feline decision tree.

See the feline decision tree in Figure 7.

Activated charcoal administration emerged as the dominant discriminator of outcome and was associated with universal survival (100% vs. 66.0% without charcoal; *p* < 0.001), indicating a markedly increased relative risk of survival and effective resolution of risk, as downstream variables did not further differentiate outcome once charcoal was administered.

In the absence of charcoal administration, outcome varied significantly. FVVF or UVVF ingestions were associated with substantially higher mortality than PVVF (23.2% vs. 11.0%; *p* < 0.01). Within this high-risk pathway, lack of consumables (products for urethral catheterization) further increased mortality (25.2% vs. 7.1%), and felines ≥9 years of age experienced nearly double the mortality of younger felines (33.1% vs. 17.1%), demonstrating that age functioned as a conditional modifier rather than a global predictor of outcome.

Among PVVF ingestions without charcoal, the decision tree demonstrated a clear stepwise gradient, with survival improving as additional intervention charges were applied. The lowest-survival feline nodes were characterized by absence of additional treatment charges (no emesis induction or hospitalization).

### 3.9 Model performance and validation

The optimized canine decision tree achieved average training and testing precisions of 0.215 and 0.217, respectively. The optimized feline decision tree achieved average training and testing precisions of 0.285 and 0.271, respectively. In both species, similar training and testing performance indicated minimal performance degradation and suggested stable, generalizable patterns rather than overfitting to the training data.

## 4 Discussion

In this large retrospective study of VVF exposure in emergency veterinary practice, we identified 32,455 probable cases, of which 31,425 were canine and 1,030 were feline, using novel regex-based data extraction methods. Canine exposures accounted for approximately 2.4% of all presentations across the dataset between 2018 and 2024. Feline cases were far less common at 0.1%, which is consistent with previous studies (1, 5, 9). These incidence figures have not been previously reported and underscore the relative rarity of VVF intoxication in felines while highlighting the value of large-scale electronic record interrogation.

Interestingly, lower body size was not associated with increased mortality and was not identified as a significant factor in decision tree analyses; in contrast, increased body weight was associated with higher mortality in canines. Since an ingested dose could not be directly measured, body size was used as a surrogate, with smaller animals presumed to receive higher relative exposure per kilogram. The absence of increased risk in smaller animals in this study suggests that relative dose may not be the primary determinant of outcome. Body surface area may represent a more accurate surrogate than body weight, given that toxicity per unit body weight can increase disproportionately with greater body mass (21), however this was also unable to be analyzed in this study. The association between increased body weight and mortality in canine cases may therefore reflect confounding factors such as age, breed-related predispositions, or comorbidities rather than a direct dose-dependent effect, and the lack of precise dose quantification limits definitive conclusions regarding dose–response relationships.

The majority of cases involved young animals, consistent with previous retrospective studies of VVF intoxication (7, 8), with a slight male predominance, likely reflecting the greater propensity of young animals to engage in exploratory ingestion behaviors (22). Among canines, smaller animals were overrepresented, likely mirroring breed popularity and relative population prevalence in the United Kingdom (23). The breeds most frequently implicated in VVF ingestion likely similarly reflect overall popularity (23), although some, such as Labrador Retrievers, are recognized for a predisposition toward exploratory ingestion (4, 12). Multi-event animals probably represent individuals with behavioral traits that increase repeated exposure risk; interestingly, these repeat events were not associated with increased mortality. These repeat exposures are likely underestimated in this study, as subsequent visits may have been assigned new case numbers and therefore not linked longitudinally. Given the small number of multi-event cases in this study, these findings should not be overinterpreted but may indicate a potential area for future research.

PVVF exposures were more common in both species, and chocolate co-ingestion was disproportionately observed in these cases, likely reflecting formulation of commercially available products. The observed December peak, particularly for PVVF exposures and chocolate co-ingestion, presumably also corresponds to seasonal product availability (15). Chocolate ingestion was uncommon overall but was analyzed as a potential confounder, given its theoretical potential to exacerbate renal injury when co-ingested with VVF (1, 19). Neither mortality analyses nor decision tree modeling demonstrated an increased risk associated with chocolate co-exposure.

Canines received more frequent clinical interventions than felines, potentially reflecting differences in presentation, clinician perception of risk, feasibility of treatment, or owner-driven decision-making (24). In felines, post-exposure interventions may have been limited by challenges associated with emesis induction (25), administration of activated charcoal, and stress related to hospitalization. It is important to note that prior emetic administration at referring practices is common and would not be captured in this dataset. Additionally, delayed presentation following ingestion may have precluded emesis in some cases.

Blood testing, fluid therapy, and hospitalization were more frequently performed in PVVF cases than FVVF cases, suggesting perceived or actual differences in clinical risk. Historically, aggressive fluid diuresis was recommended to prevent kidney injury (5, 6). More recent evidence emphasizes the risks of excessive fluid therapy—including endothelial glycocalyx disruption, hypervolemia, and edema—leading to recommendations for maintenance-level or no fluid therapy in asymptomatic, euvolemic patients (26). Despite most VVF exposures falling into this low-risk category, data did not demonstrate a clear shift toward conservative management, with clinicians continuing to prescribe fluid therapy, presumably influenced by ongoing VPIS guidance (5). Although exact volumes were unavailable due to reliance on billing records, fluid administration served as a practical surrogate for adherence. Only 2,932 cases (9%) aligned with VPIS recommendations, with no associated difference in outcome.

Furosemide use, more common in felines and fatal cases, was analyzed due its likelihood of reflecting oliguric or anuric AKI and thus serves as a surrogate marker of severe renal compromise (27, 28). However, furosemide is also used in the management of fluid overload and cardiac disease (28), so its association with mortality may in part reflect confounding by these indications rather than AKI severity alone. Consumable and procedure charges associated with urethral catheterization, typically reserved for more severe AKI (27), were also found to be associated with increased mortality risk in this study, and, unlike furosemide, are less likely to reflect confounding by unrelated indications.

The most common concomitant treatments included analgesia, antibiotics, and gastroprotectants, none of which are routinely indicated for uncomplicated VVF exposure (1). Their use may reflect concurrent conditions that cannot be identified within the design of this study or variability in clinician practice patterns. These findings raise concerns regarding antimicrobial stewardship (29) and unnecessary gastroprotectant use (30), both of which carry potential adverse effects and financial costs.

Mortality was found to be low in canines, consistent with previous reports of largely asymptomatic VVF exposure (3–10), but was significantly higher in felines, an outcome not previously reported and suggestive of possible species-specific vulnerability. Prior mortality estimates vary considerably depending on whether cohorts include all VVF exposures or only those with AKI. Among dogs with AKI, mortality has reached 50% (2/4) (3) and 46.5% (20/43) (4), whereas mortality in unselected VVF cohorts ranges from 0% (0/606) (8) to 8.3% (15/180) (5), with a pooled 24-study review reporting 5.8% (116/2,006) overall (10). Feline-specific data remain limited to two studies, one reporting 0% mortality among 13 cats (9) and the pooled review (10), which did not separate species.

Mortality outcomes in this study did not distinguish euthanasia from unassisted death, nor capture illness severity, limiting interpretation. Financial considerations may have influenced treatment intensity, particularly in felines (24), so the relative contributions of economic versus biological factors cannot be disentangled. These findings warrant further investigation into species-specific susceptibility, metabolism, and clinical progression, though lower feline case numbers may limit statistical power.

This study is the first to demonstrate that FVVF exposures were associated with increased mortality risk in both species. While this may reflect greater intrinsic toxicity, it may also relate to comparatively fewer interventions, as PVVF cases received more intensive treatment. Age was also associated with mortality in both species, potentially reflecting financial limitations, reduced pursuit of aggressive care, concurrent comorbidities, or true age-related susceptibility to VVF toxicity.

Decision tree modeling provided clinically interpretable insight into how management pathways interact with patient factors to influence outcome, while also highlighting the predominance of decontamination over more intensive downstream therapies. The strong concordance between mortality analyses and tree structure supports that these splits reflect meaningful clinical relationships rather than statistical artifact.

In canines, decision tree architecture demonstrates that decontamination was the variable most strongly associated with risk, with downstream modifiers showing little additional influence once decontamination had occurred. Age, ingestion type, and other demographic variables only became prognostically relevant when protective interventions were absent. This pattern suggests that many subsequent treatments may offer limited incremental benefit when timely decontamination has already been achieved. Age and ingestion type became prognostically relevant primarily in the absence of intervention, indicating that these factors function as vulnerability modifiers rather than independent drivers of mortality.

Importantly, additional treatment charges such as fluid therapy and blood testing appeared within higher-risk branches of the tree, but largely as markers of cases in which decontamination had not occurred. Survival frequently improved stepwise as interventions accumulated; however, this gradient was most apparent in animals already stratified into higher-risk pathways. These findings imply that additional treatments may compensate for missed decontamination but does not confer clear additional survival advantage when decontamination has been successfully performed.

In felines, activated charcoal was the variable most strongly associated with survival, potentially reflecting the practical limitations of emesis in this species (25). As in canines, once charcoal was administered, downstream variables did not further stratify mortality risk. Age and ingestion type gained prognostic importance primarily when charcoal was not given, reinforcing the concept that timely gastrointestinal decontamination was associated with a markedly lower mortality and a correspondingly reduced apparent influence of intrinsic patient factors on outcome.

This study leverages a large, standardized dataset from a single emergency provider, strengthening case capture and reducing misclassification relative to smaller, heterogeneous datasets. Differences between phone call records and clinical documentation suggest underreporting or variability in record-keeping, underscoring the value of integrating multiple data sources. Concordance between mortality analyses and decision tree structures supports the validity of regex-based case identification.

Although absolute average precision values for the decision trees were modest, this was anticipated given the highly imbalanced outcome distribution, particularly in canines. Average precision, emphasizing correct identification of rare mortality events, is more informative than overall accuracy in this context. Similar performance between training and testing datasets supports robustness of the identified pathways. Higher precision in felines likely reflects greater baseline mortality and stronger species-specific signal. Overall, the decision trees functioned as interpretable tools for risk stratification and hypothesis generation rather than high-discrimination predictive models.

This study has several important limitations inherent to its retrospective observational design. As data were derived from EHRs collected for clinical rather than research purposes, exposure classification relied on documentation within free-text clinical narratives. Although the regexes demonstrated excellent performance during validation, misclassification of exposure type FVVF to PVVF remains possible. The introduction of the UVVF exposure category mitigated, but did not eliminate, this risk. Furthermore, ingestion was frequently suspected rather than confirmed, and the quantity ingested, timing relative to presentation, and degree of mastication or processing could not be determined with this study. The absence of dose and timing information is particularly important given the unknown minimum toxic dose and the potential dose-dependent nature of toxicity.

Mortality was used as the primary outcome measure; however, death included both unassisted death and euthanasia. Euthanasia decisions may be influenced by perceived prognosis, comorbidities, financial constraints, or owner preference, introducing non-biological determinants into the outcome (24). Additionally, AKI was not confirmed, limiting the ability to directly attribute mortality to renal injury secondary to VVF exposure. As such, mortality should be interpreted as a clinically relevant but imperfect proxy for severe toxicity.

Treatment variables require cautious interpretation. Interventions such as emesis induction, activated charcoal administration, fluid therapy, and hospitalization were not randomly assigned but determined by clinician judgment, perceived severity, and timing of presentation. Consequently, confounding by indication is highly likely. Apparent protective associations identified in both statistical testing and decision tree models may therefore reflect earlier presentation, lower initial severity, or greater opportunity for intervention rather than direct causal treatment effects. The study design does not permit causal inference regarding therapeutic efficacy.

Decision tree models, while useful for identifying structural patterns within large datasets, have additional limitations. Trees identify splits that maximize statistical information gain and do not establish biological or causal relationships. Early splits based on treatment variables likely encode clinical workflow and triage behavior as much as pathophysiological risk. Although internal validation demonstrated similar training and testing performance, average precision values were modest, particularly in canines where mortality was rare. Rare-event prediction can produce unstable minority-class performance, and small absolute differences may generate large relative risk estimates. Furthermore, repeated ingestion events were retained in model development to maximize available information, which may introduce correlation between observations and influence tree structure. The models were validated only within a held-out subset of the same network and have not undergone external validation; therefore, generalizability to other practice settings, geographic regions, or veterinary care systems is unknown.

Residual confounding is also likely. Important clinical variables including baseline renal parameters, comorbid disease, severity of clinical signs, and precise time-to-treatment were unable to be determined with this study design. Without these factors, the models cannot fully adjust for underlying illness severity. Nodes demonstrating high mortality may represent relatively small subgroups, and such terminal leaves may be sensitive to small data changes despite hyperparameter optimization.

Finally, although this study represents one of the largest cohorts of VVF exposure to date, feline case numbers were substantially lower than canine cases. The higher observed feline mortality, while statistically robust within this dataset, should be interpreted cautiously given smaller absolute numbers and potential species-specific differences in presentation or management.

Taken together, these limitations underscore that the findings should be interpreted as hypothesis-generating associations within a large real-world dataset rather than definitive evidence of causal toxicity mechanisms or treatment efficacy. Prospective studies incorporating standardized exposure assessment, dose quantification, AKI staging, and temporal treatment data are required to clarify causal relationships and refine clinical recommendations. Detailed evaluation of high-mortality cases may identify modifiable risk factors to inform refined management algorithms.

In conclusion, this study demonstrates that regex-based data extraction can identify a substantially larger cohort of VVF exposure cases than previously reported, enabling detailed comparison of FVVF and PVVF ingestions in canines and felines. VVF exposure is common in canine emergency practice and less frequent but associated with substantially higher mortality in felines. FVVF was consistently associated with greater mortality risk than PVVF ingestion in both species, while increasing age functioned as an important vulnerability factor. Gastrointestinal decontamination emerged as the strongest predictor of survival among the variables analyzed, with markedly lower mortality observed when treatments were performed. Based on these findings, management of suspected exposure should prioritize prompt assessment and decontamination where clinically appropriate followed by individualized supportive care guided by clinical status rather than exposure type alone. In asymptomatic, euvolemic patients, conservative or absent fluid strategies may be appropriate, whereas older animals and those ingesting FVVF may warrant closer monitoring and escalation of care. Prospective studies are required to define toxic thresholds and refine evidence-based treatment protocols, particularly in felines.

## Data Availability

The raw data supporting the conclusions of this article will be made available by the authors, without undue reservation.
