# Supplementary material for: A multicenter, retrospective, cross-sectional analysis of fresh and processed Vitis vinifera (grape) ingestion in dogs and cats
Source: Front Vet Sci. 2026 Aug 19;13:1812885. doi: 10.3389/fvets.2026.1812885 (PMC13533654; doi:10.3389/fvets.2026.1812885)
Supplement: Supplementary file 1 [file Supplementary_file_1.docx]

# **Supplementary Material – Regular Expressions**

**Fresh *Vitus Vinifera* Regular Expression**

(?<![Hh]x of eating )(?<![Hh]istory of eating )(?<![Ss]ize of a medium )(?<![Ss]ize of a small )(?<![Ss]ize of large )(?<![Ss]ize of medium )(?<![Ss]ize of small )(?<![Ss]ize of a large )(?<![Ss]ize of a )(?<![Bb]igger than )[Gg]ra[p]?[o]?e(?![\\-]?[\\s]?size)(?!fruit)(?!s lepto). (?<![Hh]x of eating )(?<![Hh]istory of eating )(?<![Ss]ize of a medium )(?<![Ss]ize of a small )(?<![Ss]ize of large )(?<![Ss]ize of medium )(?<![Ss]ize of small )(?<![Ss]ize of a large )(?<![Ss]ize of a )(?<![Bb]igger than )[Ff]ra[p]?[o]?e(?![\\-]?[\\s]?size)(?!fruit)(?!s lepto). (?<![Hh]x of eating )(?<![Hh]istory of eating )(?<![Ss]ize of a medium )(?<![Ss]ize of a small )(?<![Ss]ize of large )(?<![Ss]ize of medium )(?<![Ss]ize of small )(?<![Ss]ize of a large )(?<![Ss]ize of a )(?<![Bb]igger than )[Gg]raoe(?![\\-]?[\\s]?size)(?!fruit)(?!s lepto). (?<![Hh]x of eating )(?<![Hh]istory of eating )(?<![Ss]ize of a medium )(?<![Ss]ize of a small )(?<![Ss]ize of large )(?<![Ss]ize of medium )(?<![Ss]ize of small )(?<![Ss]ize of a large )(?<![Ss]ize of a )(?<![Bb]igger than )[Gg]r[e]?a[e]?p(?![\\-]?[\\s]?size)(?!e size)(?!esize)(?!h)(?!fruit)(?!s lepto). (?<![Hh]x of eating )(?<![Hh]istory of eating )(?<![Ss]ize of a medium )(?<![Ss]ize of a small )(?<![Ss]ize of large )(?<![Ss]ize of medium )(?<![Ss]ize of small )(?<![Ss]ize of a large )(?<![Ss]ize of a )(?<![Bb]igger than )[Gg]rapa(?![\\-]?[\\s]?size)(?!fruit)(?!s lepto). (?<![Hh]x of eating )(?<![Hh]istory of eating )(?<![Ss]ize of a medium )(?<![Ss]ize of a small )(?<![Ss]ize of large )(?<![Ss]ize of medium )(?<![Ss]ize of small )(?<![Ss]ize of a large )(?<![Ss]ize of a )(?<![Bb]igger than )[Gg]reape(?![\\-]?[\\s]?size)(?!fruit)(?!s lepto).

**Processed *Vitus Vinifera* Regular Expression**

(?<!no )(?<!did not contain )(?<!did not contain any )[Rr]a[i]?[s]+[i]+n[i]?(?!g). (?<!no )(?<!did not contain )(?<!did not contain any )[Rr]a[i]?[s]+[a]+n[i]?(?!g). (?<!no )(?<!did not contain )(?<!did not contain any )[Rr]a[i]?[s]+[e]+n[i]?(?!g). (?<!no )(?<!did not contain )(?<!did not contain any )[Rr][i]?a[s]+[i]+n[i]?(?!g). (?<!no )(?<!did not contain )(?<!did not contain any )(?<![Bb]lack)[Cc]u[t]?[r]+a[n]+t(?!ly). (?<!no )(?<!did not contain )(?<!did not contain any )(?<![Bb]lack)[Ff]u[r]+ant(?!ly). (?<!no )(?<!did not contain )(?<!did not contain any )(?<![Bb]lack)[Cc]i[r]+ant(?!ly). (?<!no )(?<!did not contain )(?<!did not contain any )(?<!did not contain any raisins)(?<![Cc]on)[Ss]ul[s]?[t]+[a]?[n]+[s]?. [Ff]ruit and nut. [Ff]urit and nut. [Ff]uirt and nut. [Ff]ruti and nut. [Tt]ruit and nut. [Ff]ruit [\&] nut. [Ff]urit [\&] nut. [Ff]uirt [\&] nut. [Ff]ruti [\&] nut. [Tt]ruit [\&] nut. [Tt]rail mix. [Tt]rail mid. [Tt]rial mix. [Dd]ried fruit. [Dd]rief fruit. [Mm]uesli. [Gg]ranola. [Ff]ruit cake. [Hh][o]?[t]?[ ]?cross bun. [Hh]ot corss bun. [Cc]hristmas c[a]?ke. [Cc]hrsti[a]?mas c[a]?ke. [Cc]hrsit[a]?mas[e]? c[a]?ke. [Cc]hristmas ckae. [Cc]hrsti[a]?mas ckae. [Xx]mas cake. [Ee][c]+les. [Ww]elsh cake. [Mm]alt loaf. [Ss]cone. (?<![Rr]e)[Ss]once. [Ss]once. [Ff]ruit toast. [Ff]ruit bread. [Bb]ara brith. [Dd]undee cake. [Ss]imnel. [Qq]ueen cake. [Tt]ea cake. [Mm]ince[d]?[ ]?pie. [Mm]icne[d]? pie. [Mm]iene[d]? pie.

**Chocolate Regular Expression**

(?<![Ee])(?<!white. )(?<!white)(?<![Nn]o. )(?<![Nn]o)(?<![Hh]x of eating. )(?<![Hh]istory of eating. )[Cc]hoc(?!king)(?!co)(?!ed)(?!olate. brown)(?!ked). [Gg]alax. [Cc]adbury. [Ee]aster[ ]?egg. [Mm]ars[ ]?bar. [Ss]nickers. [Dd]airy[ ]?milk. [Tt]obler. [Mm]ilka. [Cc]ocoa. [Cc]acao. [Mm]alte[a]?ser. [Mm]?[Bb]?illionaire shortbread. [Mm]ilky bar. [Mm]instrel. [Aa]ero(?!phag). [Ff]e[r]+e[r]+o. [Kk]it[ ]?kat. [Tt]hornton. Truffl. Twix. [Yy]orkie bar. [Bb]ounty. [Bb]oost bar. [Rr]ipple. [Tt]wirl. [Bb]ueno. [Dd]ouble decker. [Ff]re[d]+o. [Dd]aim. [Hh]ershey. [Ll]indt. [Rr]eeces. Smartie. [Ll]ion bar. [Gg]uylian. (?<![Ss])[Cc]runchie. [Rr]evels. [Rr]itter(?!ia). [Rr]ocky road.
